# Covid-19: The aftermath for orthodontics

**DOI:** 10.1590/2177-6709.25.2.007-008.edt

**Published:** 2020

**Authors:** Flavia Artese

**Affiliations:** 1 Universidade do Estado do Rio de Janeiro, Departamento de Odontologia Preventiva e Comunitária (Rio de Janeiro/RJ, Brazil).

As early as 2007, a paper published in the Clinical Microbiology Reviews on SARS coronavirus infection already warned us that *“the presence of a large reservoir of SARS-CoV-like viruses in horseshoe bats, together with the culture of eating exotic mammals in southern China, is a time bomb”*.^1^ In spite of this grave alert, we are currently experiencing one of the most serious pandemias in human history. I believe most of us are still in absolute dismay with this disease, which was first detected in hospitals in Wuhan and probably originated in the Huanan seafood market in China.^2^ Due to the high degree of contamination of this virus, the world came to a halt or at least 1/3 of it. In fact, the world is scared. So much so that leaders in the world seem to be lost, divided in their roles of great responsibility, between saving lives due to the corona virus or focusing on the consequences of the economic lag that will follow due to social isolation. 

Unprecedented amount of information piled our smartphones and mailboxes with so many divergent opinions, since never such a catastrophic event had happened after the communication revolution. So much information makes it quite difficult to separate the wheat from the chaff, which increases anxiety and blurs our judgement on what will be best for society. Scientific journals are making a huge effort in publishing, as quickly as possible, the available current knowledge on this virus, which seems to grow faster than our capability of retrieving clinically applicable information. 

An excellent summary of information by the Director of the NIH^3^ explains very clearly why social distancing may be the best immediate solution to slow the spread of COVID-19. For every confirmed case of this disease there are likely another five to ten people with undetected infections. These individuals represent the source of 86% of the infections. According to the Imperial College of London, in a modelling study^4^, it was estimated that: if nothing was done, 510,000 people would have died in the UK; with vertical isolation methods, 250,000 deaths would be expected, and with strict social isolation, there would be an estimate of 20,000 deaths. So, staying at home and reducing social contact may allow society to prepare its health-care systems and consequently reduce death tolls. This measure will also mitigate damage while researchers have more time to develop treatments and most specially a vaccine, which would take approximately 12 to 18 months to be approved for human use.^3^

Science has recently and very rapidly been considered by leaders and society as our main hope. Holden Thorp, editor-in-chief of Science Journals, couldn’t have written a better editorial^5^ which echoed the voices of world researchers. He described how government priorities do change when facing fear and desperation, when the President of the USA asked the scientific community to do him a favor and speed things up in preparing a vaccine or a treatment for COVID-19. Quite a contradiction when research grants have been reduced and an antivaccine task was proposed in the USA. Brazilian government also walked this same path, regarding research grants and student stipends.

This social shut down has also a yet unmeasured effect on the economy with different impacts. Countries such as my own, Brazil, which was emerging from an economic crisis, will have an enormous set back in its economy. People will also suffer from this indirect consequence of the pandemia. But, staying at home has changed our society in many aspects. 

We are fortunate to have technology on our side and use at its most. For example, internet communication allows home schooling for all levels of education. Huge conferences were cancelled, but some were transformed into virtual lectures, such as the AAO Annual Session. Museums, theaters, ballets and operas are being offered freely in the virtual world to entertain those who are in lockdown. Likewise, scientific journals opened their resources to allow professionals to access information at their need. The Dental Press publishers also joined this movement, opening their complete digital data, called Dental GO, and offering dentists all over the world an access to a collection of over 6,000 publications in Dentistry, which include the *Dental Press Journal of Orthodontics*.

The impact in Dentistry is incalculable. All sorts of discussion have risen mostly regarding our possibilities to see and treat our patients, since Dentistry is considered one of the professions with the largest risk of contamination. Droplets and aerosols are generated in a dental clinical setting, and even standard protective measures are not effective enough to prevent the spread of COVID-19.^6^ Leading professionals have proposed an increase in the use of digital orthodontics and the management of patients through “teledentistry”. In fact, a whole new world seems to sprout from the lockdown despair and the economic impact our profession will go through. I have been hearing so many opinions, which in my point of view, at this moment, are merely ideas which we try to grab as a lifeline.

The only certainty I have right now is that mankind will emerge from this pandemia with profound scars. Life will not be the same. But, if I may also place my humble ideas of the future, I think that our extreme value and dependence on technology in our current age, may gain a more weighted application. Mankind is being reminded that nothing replaces human touch, especially when you are in agony. Curing is also dependent on feelings, on trust, on exchanging glances, and machines or artificial intelligence is a mechanism of aid and not of substitution of our most precious characteristic: humanity. When our doors open again, we will be thirsty for gatherings, for embraces. Students will be longing for presential classes and patients will be wishing to be seen by their doctors. And, maybe science will be adequately valued by government administrations. I also hope that, as an aftermath, orthodontics will regain its place as a medical profession and not be inconsequently sold over the counter or in shopping malls.

With a heart and mind full of hope, I wish you good readings.
